# Progressing Towards a Human-Centric Approach in Cancer Research

**DOI:** 10.3389/fonc.2022.896633

**Published:** 2022-07-19

**Authors:** Aditya Parekh, Subhayan Das, Chandan K. Das, Mahitosh Mandal

**Affiliations:** ^1^ School of Design, Anant National University, Ahmedabad, India; ^2^ Genetics and Development, National Centre For Biological Sciences, Bengaluru, India; ^3^ School of Medical Science and Technology (SMST), Indian Institute of Technology, Kharagpur, India; ^4^ Cancer Biology, University of Pennsylvania, Philadelphia, PA, United States

**Keywords:** drug attrition, human-centric models, cancer research, drug discovery, animal-centric models

## Abstract

Despite the advancement in research methodologies and technologies for cancer research, there is a high rate of anti-cancer drug attrition. In this review, we discuss different conventional and modern approaches in cancer research and how human-centric models can improve on the voids conferred by more traditional animal-centric models, thereby offering a more reliable platform for drug discovery. Advanced three-dimensional cell culture methodologies, along with *in silico* computational analysis form the core of human-centric cancer research. This can provide a holistic understanding of the research problems and help design specific and accurate experiments that could lead to the development of better cancer therapeutics. Here, we propose a new human-centric research roadmap that promises to provide a better platform for cancer research and drug discovery.

## 1 Introduction

Cancer arises when tumor cells start invading nearby tissues, leading to the disruption of tissue homeostasis. Common treatment strategies against cancer include surgery, chemotherapy, radiotherapy, and immunotherapy, depending upon the stage and the nature of cancer. Currently, every sixth person in the world dies of cancer, which is second only to the cardiovascular diseases (1). Despite advancements in early detection and novel therapeutics, cancer is still the second largest cause of human death (1). Globally, around 19.3 million new cancer cases were estimated, and 10 million deaths for the year 2020 (2). This is primarily attributed to our lack of understanding of the complexity of the disease. Some of the common complexities arise from heterogeneity within the cancer tissues and the development of therapeutic resistance during and after the treatment (3–6). Cancer is a genetically heterogeneous disease; i.e. cellular heterogeneity exists within a tumor population. Tumor heterogeneity suggests that it contains more than one cell types, which exhibit differential ability to proliferate, migrate, maintain stemness, and response to therapy (4). The stem cell-like population within the tumor are inherently more resistant to therapy [due to over-expression of specific drug neutralizing/exporting proteins, and anti-death proteins (7, 8)] and less accessible to drug molecules, whereas simultaneously also contributing to tumor heterogeneity. Therapy resistance is the result of either over-proliferation of cell types that are inherently resistant or may be acquired progressively during treatment. The sub-population of cancer cells generally coexist with immune and other non-cancerous cells in a complex ecosystem called tumor microenvironment. In addition to the intra-tumor cellular heterogeneity, the cancer cells co-exist with different non-cancerous cells like the Cancer associated fibroblasts (CAFs), endothelial cells, and immune cells (like macrophages, microglia, and lymphocytes); and the non-cellular components like extracellular matrix components (ECM) (fibronectin, laminin, collagen, hyaluronan, integrin etc.) to form a complex three dimensional (9) ecosystem called tumor microenvironment(TME) (5, 10). Different cells in the TME can cross-talk and release factors which can modify ECM. The chemical and physical signals triggered by ECM have been shown to regulate the tumor progression and therapeutic resistance by modulating cancer heterogeneity, clonal evolution, epithelial-mesenchymal transition, invasion, migration, neovascularization, and metastasis (9, 11, 12). Remodelling of endothelial cells, cross-talk between CAFs and tumor cells, and paracrine signalling by Tumor associated macrophages, all as a part of TME have been shown to promote cancer progression (13). Furthermore, intra-tumoral mechanisms of metabolite communications between different cell types act in a symbiotic way to promote tumor metabolism, maintenance and growth (14). *In the last few years, researchers have discovered that TME mediate therapeutic resistance by regulating drug availability *(13) *and interstitial fluid pressure *(15). Because of the complexities, even combinational therapy is unable to kill all the cancer cells, leading to disease relapse. Together, these factors play a pivotal role in tumor progression and therapy resistance (4–6, 16, 17), thus making it challenging to design targeted therapy. However, recent developments of the three dimensional (3D) *in vitro* cell culture methodologies and platforms provide immense scope to exploit the biology of tumor through better mimic of the TME (18, 19).

Despite substantial funding in research and development globally, less than 85% of the drugs showing encouraging signs in the pre-clinical cancer models succeed in the early clinical trials. and less than half of those passing through Phase 3 clinical trials make it for licencing (20). In fact, roughly 1 in 10,000 pre-clinical compounds reaches the market (21). Because of such a low success rate, prices of anti-cancer drugs touch an unaffordable range. There are many anticancer drugs at present which cost more than $10,000 (per year per patient) and the supporting claim pharma companies make to justify these costs is the high drug attrition rates at phase 2 and phase 3 of clinical trials (21–23). One of the main reasons for high cancer drug attrition is their reduced efficacy in humans compared to animal models (21, 23). This points out our lack of understanding of the complexity of the disease, which may be due to the way chemotherapeutic drugs are tested preclinically before being brought in the purview of the clinical trial. Heavy dependence on animal models for pre-clinical studies is perhaps one of the important reasons for the current setback. A better understanding of drug dynamics inside the human body and their interaction with different cell types will help decrease the intensity of the problem in hand. Thus, it becomes essential not to consider cancer from only tumor cell-centric perspective but rather as an ecosystem containing a plethora of cells, acting as a protective shelter for tumor cells. The use of human-centric models may help in mitigating the limitations posed by animal-centric research models. Its cumulative understanding will be useful to go beyond our current limitations and prepare new generation drugs with better efficacy. Traditional ways of performing preclinical research primarily depend on human cell lines (2D *in vitro*), and non-human animals, such as rodents and primates (*in vivo*). Historically, animal-centric research has been heavily relied on for understanding cancer and testing new anti-cancer drugs because of the conservation of basic biological principles and the evolution of modern genetic tools. Unfortunately, the traditional animal-based strategies for cancer research have not been able to deliver as per the expectations. Animal research has its own limitations and has not been the best of the approaches to depend upon as it fails to reliably mimic human cancer models, leading to high drug attrition rates (21, 24). In recent years, there has been an increased focus on developing more human-centric methods for enhancing drug predictivity and ensuring human-predictive drug efficacy. Due to the known limitations of the existing systems of drug-testing, it becomes essential to explore varieties of model systems relevant to humans, including human-specific models for improving drug efficacy, reducing drug costs, and enhancing quicker market reach.

In this review, we summarize the current and traditional ways of cancer research, including 2D cell culture and the use of various animal models. Thereafter, we look upon the avenues and opportunities which are presented by the advanced human-based cancer models such as modern 3D (three-dimensional) culture, organ-on-a-chip, systems biology approaches, and *in silico* models, which promise to go past the limitations posed by the animal-centric research models. We further discuss a future roadmap of cancer research, which focuses on the application of different advanced human-centric models and explores their scope to replace animal models.

## 2 Evolution of Cancer Research Models

### 2.1 *In Vitro* Models

The traditional *in vitro* cell culture models use two dimensional (2D) techniques, employing isolated immortal cell lines originally obtained from people with cancer (25) and now banked and commercially available. The first cell line to be established was the HeLa cell line, which was obtained in 1951 from Henrietta Lacks, a 31yr old cervical cancer patient (26). 2D models are still largely used for many experiments that involve deciphering molecular mechanisms and drug discovery. However, there are limitations with these models, leading to partly accurate results and predictions. Although the 2D cell culture model does not resemble the actual tumor and overlooks the complex cellular architecture of cancer cells and tumor microenvironment, it is still the easiest and most economical way to perform large scale screening and pilot studies. In real world. cell division, maintenance of cellular homeostasis, and cell death all occur in a three-dimensional environment within the complex tissue architecture in higher organisms. Experiments with cells growing on a two-dimensional plastic surface cannot mimic the inherent intricacies of cell-cell interactions, cell-matrix interactions, and biomolecule availability and drug dose response (hormesis) (27). This led to the development of different techniques, trying to mimic the 3D tissue microenvironment, and these 3D approaches are being actively used to study diseases like diabetes (28, 29), cardiomyopathy (30), and cancer (31). Culture of cells in 3D, both with and without scaffolds, is revolutionizing biological research (32). The first research with cells in 3D dates to more than a century back when bacteria were clamped in a hanging drop (33). Later, this technique was employed to grow the first tissue in a scaffold-free manner (34). 3D cell culture models relevant to human disease started coming to the limelight since the 1980s and have been advancing since then. 3D cancer models resemble the tumor architecture more closely (35) and can mimic the cellular crosstalk in the different co-culture systems (36). Different cancer and non-cancer cell lines are grown in 3D scaffolds to resemble tumor microenvironment. Cytotoxicity testing, modelling of cancer stem cells, and epithelial to mesenchymal transition is better represented in the 3D culture system when compared to conventional 2D culture (31, 37). However, the major limiting factors of scaffold-dependent 3D cell culture models are its inability to mimic the biomolecular circulation and the controlled availability (which is physiologically aided by vasculature). Further, these scaffold-dependent models fail to mimic the appropriate biophysical extracellular structures/cues as necessary to tissues. To date, conventional 2D and 3D *in vitro* models fail to fully replicate the complex dynamic system operating within a tumor (38).

Other notable advancements in cancer research are the organoid and spheroid 3D cell culture systems. Both Spheroid and organoid models are 3D cell-culture models but with marked differences. A spheroid model is the simpler of the two, formed by of simple 3D aggregation of cells and does not contain any extracellular matrix or hydrogel scaffold and thus cultured as free floating aggregates. It can be developed from any cancerous or non-cancerous cell line or primary cells (by using a non-adherent plate). On the other hand, an organoid is a complex 3D structure of cells which is often supported by extracellular matrix or hydrogel scaffold and specific growth factors (32, 39) and is developed using stem cells primarily derived from the human healthy to tumor tissues. The cells in the spheroid predominantly has stem cell like property but, an organoid contains more differentiated cells (aided by scaffold and growth factors) and closely resembles the tissue architecture and function (40), which can be cryopreserved (41). Organoids grown with this method acquire a micro-anatomy, which is similar to the native tissue by self-organization and spatial orientation (42) and helps retain heterogeneity of the original tumor (19). Patient-derived organoid models have been generated with liver, colorectal, pancreatic, and prostate cancer (19). Gene-editing to introduce mutation and co-culture techniques to mimic different cellular interactions have also been recently possible using organoid models in cancer (43). The organoid models have advantages over the regular 3D model in simulating tumor-specific differential growth patterns like quiescence. On the other hand, the spheroid culture presents another 3D model, which resembles the 3D structure of a tumor and is established with low technical difficulty and cost. These spheroids loosely resemble the architecture of the native tumor and can also be co-cultured with other cells to mimic the exact tumor microenvironment (44). They have been shown to be more reliable for *in vitro* drug screening (45) and for modelling drug-resistance (46) than conventional 2D culture.

Features like hypoxic gradient and nutrition gradient can be mimicked more realistically in these advanced 3D models in comparison to the native tumor (47). Over the years, with the advancement in the knowledge of stem cells and the usage of patient-derived tumor organoid models, these advanced 3D models hold key promise in the field of personalized medicine (48–50). These characteristics make them more relevant compared to *in vivo* animal models, being not only cheaper but also lacking the ethical conflicts of the latter (51). Recently, there has been significant progress in the application of microfluidic-based devices to create models of human organs, specifically for drug development and bio-modelling. This is called the organ-on-a-chip/organ chip model, which accounts for the constant circulation of media, mimicking the human vascular system. Scientists have been successful in developing organ-chip models of intestines, blood-brain barrier, bone marrow, lungs, liver, kidney, and skin and using them as effective preclinical models in cancer, and other diseases (52). The organ chip model recapitulates tissue-tissue interface, and multicellular architecture very closely and helps modulate local molecular, chemical, and biophysical parameters in a precisely controlled manner (53). Tumor-chip models have been shown to closely resemble tumor stroma and are beneficial for modelling multi-organ metastasis (19). The organ-on-a-chip model has been used to study important cancer hallmarks like angiogenesis, migration, and invasion in a precise manner (53). Further, 3D based microfluidic research shows applications in the study involving drug testing, metastasis, and drug resistance (54). Although organ-chips are by far the most advanced of the *in vitro* technology systems, and successfully mimic organ-level physiology, this technology is still in its infancy and is likely to require more validation in different contexts with *in vivo* human system before we move to a paradigm shift in our methodologies for basic and translational cancer research.

### 2.2 *In Vivo* Models

Rodents are the main animal used as *in vivo* models to test drug efficacy. The xenograft mouse model is one of the most prominently used mouse models to model cancer and test drugs in which in most cases commercial human cancer cell lines are inserted within the immunocompromised mice (55). This strategy has been perceived as the gold standard for the preclinical drug testing and elucidating molecular mechanisms in cancer. However, these animal models have significant limitations. Since the model organisms are not genetically identical to humans, the tumors do not reflect the exact human condition and thus makes it difficult to predict drug efficacy and behavior. Furthermore, because the immune system is compromised, complex cellular interactions between the immune cells and the cancerous cells cannot be studied. Considering that we now know the role of immune cells in cancer cell survival and progression (56), these might not be the appropriate way to model human cancers (55). Furthermore, these cells which have been grown for many generations in plastic, may not be very ideal to recapitulate tumor close to being original. More recently, animal models have been used for patient-derived xenograft models (PDX) and genetically engineered models (GEM). In the PDX models, the primary tumor cells are taken directly from patients to develop a tumor in immunocompromised mice. It has added advantages of mimicking the heterogeneity of parental cancer. However, it also has the disadvantage of using immunocompromised animals (57) and that patient-derived cells have been shown to undergo animal-specific modifications (58), besides being expensive and time consuming. There have been several attempts to develop GEM to mimic the original human tumors (59). The mutations are either made in somatic or germline cells to model the tumor. Recent progress in gene editing tools has advanced this field considerably. These models are supposed to be superior to conventional models (59). However, the major problem of these models is that they do not incorporate human cells, and they are not cost-effective. An alternative to rodents, invertebrate (*Drosophila*) and fishes (zebrafish) have been used less prominently as a model for therapeutic research because of their lesser resemblance to the human genome. Naturally occurring tumors in canines and felines, are also used in cancer research. Although they have the advantage of naturally occurring tumors (60) and longer life span when compared to rodents, they have their own limitations. They do not exactly mimic human physiology. Additionally, because of the lack of scope of genetic modifications and the extra demand for time and efforts, lack of sufficient numbers, these models do not promise to be the most ideal for cancer research (61).

## 3 Emerging Human-Centric Research Models

### 3.1 Human Cell-Based Models

Besides the widely used traditional cell culture and animal models, human-specific models for cancer research offer exciting avenues for preclinical testing. Human tissues have been used in oncology research for a long time (62). The ongoing advancement in tissue and cell culture reveal their promise for overcoming the common problems of animal-based research. One promising aspect is the primary cell culture from patient samples, where tumors from the patients are directly used and cultured on a dish, maintaining the inherent cellular heterogeneity. This technique is more suitable for drug testing and modelling chemoresistance (63). In comparison to conventional scaffold-based 3D cell culture models, the spheroid and organoid models of the primary tumor are far better representative of the original human tumor and can be used for better cancer modelling. A more recent trend is to utilize the tumor cells in a co-culture system with other cells of the tumor stroma, such as fibroblast and immune cells. These models can be used for high throughput drug-screening assays and have been successfully tried in a 96-well culture system (45, 64, 65). Alongside, organ chip model has been successful in taking care of fluid circulation and biomechanics of the tissues very closely. These microfluidic devices perhaps provide the best platforms along with the advanced 3D models to build a better and more complete cancer model which can closely resemble the parental tumors (53, 66). The most advanced form of human tissue reconstruction is 3D bioprinting which is now applied to model different types of cancer. This field combines the principles of biomaterials and tissue engineering with the delicacy of cell biology to provide a printable tissue that can be used for organ transplantation, drug discovery, and personalized medicine (67). These models have shown promising results in mimicking native tissue microenvironment and complexities (68–70). 3D bio-printed cancer models showed greater cell survival, protein expression patterns that resemble host cancer, and higher chemoresistance to anticancer drugs similar to host. They can also retain features that closely resembled host tumor characteristics like tumor heterogeneity, necrotic cores, and microenvironment. These methods have helped to better understand cancer formation, progression, and response to anticancer therapies. Since animal models differ physiologically from human cancer, a bio-printed cancer model with human cells can be a better representative. These models are also able to mimic the effects of metastasis which is a significant advancement in the fields of *in vitro* cancer modelling. Bioprinted cancer models have been shown to provide effective reconstructions of host cell and cell-matrix interaction patterns. These models were not only able to simulate metastasis, but the local chemical signalling profile was also found to be similar to the host. The current limitation of this technique is that the cells can only be cultured for a limited period. The expansion of human cell biobanks will help in the supply of a variety of patient’s cancer cells (of different grades and types) to research institutes, which currently have limited accessibility. We discussed this further in the last section (Future roadmap and perspective).

### 3.2 Computational Cancer Models

Data generated from OMICs-based research approaches (such as genomics, transcriptomics, proteomics, and metabolomics) have added greater value to the field of cancer research as it has in any other biomedical field. The ‘OMICs’ generally refers to the analysis of structure, function, and origin of biomolecules derived from high throughput studies, along with their implication in the applied fields of biology (71). OMICs based studies tend to generate vast amounts of data, and a systematic approach is needed to store, access, and analyse them using various tools. Advances in the field of molecular and computational biology have enabled us to perform genomic, proteomic, and transcriptomic analyses from the patient samples or primary cell culture (72, 73). Databases generated from “Omics” studies certainly provide a platform for biologists to access them from different sources and integrate it in their research (74, 75). Although some of these databases are behind a paywall or require specific permission, most of these are in the public domain and are made freely available for cancer researchers. Some of the publicly available database document information regarding the human disease, mutations, expression of proteins, and response to drugs (e.g., TCGA Database) while other databases enlist similar information regarding cell lines (e.g., CCLE). Databases are also available to explore other potential areas like epigenetic modification, miRNA regulation, and detailed mutation analysis (76). Some of the relevant databases are listed in **Table 1**, nevertheless describing them in detail is beyond the scope of this article. As we have discussed earlier regarding the therapeutic challenges posed by tumor heterogeneity, drug resistance, and drug mistargeting, it becomes vital to analyse OMICs data to understand disease progression, identify molecular markers, and response to therapy (98). The main use of cancer databases is to enable the generation of scientific hypotheses from the available datasets. These hypotheses can vary from the expression of a certain gene to the efficiency of certain drugs. In certain cases, these hypotheses can be tested using scientific tools, like cBioportal, which gives information about associated mutations, responsible protein function, and their association with survival. Alongside, proteomic databases are being used for developing diagnostics, identifying mechanisms, and designing treatment strategies. The main purposes of these databases are to save time by providing information that might be acquired from traditional wet lab and animal experiments, whereby avoiding repetition and duplication of experiments Moreover, many of these databases are based on integrated human and cell line experimental data and are carefully curated. Therefore, these databases can provide a starting platform where we can get information similar to that obtained with animal models and help to avoid further or duplicate use of animals. With data being generated at a high pace and their huge scope of application, one must admit that above-mentioned databases have been underutilized and can be used more broadly and efficiently.

**Table 1:** Major databases used in cancer research.

Type	Name	Main features	Type of available data	Weblink	References
**Proteomics**	Cancer-HPP (The Human Cancer Proteome Project)	Characterize proteomes, proteome forms, and protein networks from different cancers	Patient and cell line data	https://www.hupo.org/Human-Cancer-Proteome-Project	(77, 78)
	CPTAC (Clinical Proteomic Tumor Analysis Consortium)	Generates both peptide-spectrum-match (PSM) reports and gene-level reports	Patient data	https://hupo.org/Clinical-Proteome-Tumor-Analysis-Consortium-(CPTAC)	(79–81)
	TCPA (The Cancer Proteome Atlas)	Diverse visualization and analysis of protein data for patient tumours and cancer cell lines	Patient and cell line data	http://tcpaportal.org	(82)
	TCGA (The Cancer Genome Atlas)	Houses huge amount of genomic, epigenomic and transcriptome data with integrated analysis platforms	Patient	https://portal.gdc.cancer.gov/	(83)
**Genomics**	COSMIC Catalogue Of Somatic Mutations In Cancer)	Largest somatic mutation database; genome sequencing paper curation	Patient and cell line data	http://cancer.sanger.ac.uk	(84)
	cBioPortal	Graphical summaries; gene alteration; processed data; visualization	Patient and cell line data	http://www.cbioportal.org/public-portal/	(85)
	GDAC (from Broad Institute)	Downstream Data analysis platform using; TCGA data giving user-friendly reports	Patient data	http://gdac.broadinstitute.org/	(86)
	SNP500Cancer	Sequence and genotype verification of SNPs	Patient data	http://snp500cancer.nci.nih.gov	(87)
	canEvolve	Comprehensive analysis of tumour profile; Data from 90 studies involving more than 10,000 patients	Patient data	www.canevolve.org/	(88)
	MethyCancer	Relationship between DNA methylation, gene expression and cancer	Patient and cell line data	http://methycancer.psych.ac.cn	(89)
	SomamiR	Correlation between somatic mutation and microRNA; genome-wide displaying	Cell line data	http://compbio.uthsc.edu/SomamiR/	(90)
	NONCODE	ncRNAs; lncRNAs; up-to-date and comprehensive resource	Patient and cell line data	http://www.noncode.org/	(91, 92)
	canSAR	Multidisciplinary information; drug discovery	Cell line data	https://cansar.icr.ac.uk/	(93)
	CGWB	Visualization; gene mutation and variation; automated analysis pipeline	Patient data	https://cgwb.nci.nih.gov/ https://omictools.com/cgwb-tool	(94)
	UCSC Cancer Genomics Browser	Clinical information; gene expression; copy number variation; visualization	Patient and cell line data	https://genome-cancer.soe.ucsc.edu/	(95)
	GDSC (Genomics of Drug Sensitivity in Cancer)	Drug sensitivity information; drug response information	Cell line data	http://www.cancerrxgene.org	(96)
	TCGA (The Cancer Genome Atlas)	Houses huge amount of genomic, epigenomic and transcriptome data with integrated analysis platforms	Patients data	https://www.cancer.gov/about-nci/organization/ccg/research/structural-genomics/tcga	(83)
**Metabolomics**	HMDB (The Human Metabolome Database)	Metabolomics database, seven cancer drug metabolism pathways and twelve cancer drug action pathways	Human data	https://hmdb.ca/	(97)

The major advantage of many of the above mentioned repositories is that many of them contain patients’ data and thus are useful for accurate, human-relevant predictions. Increasingly, more data sets are being validated using other supporting experiments and have slowly started getting global acceptance. These approaches have the potential not only to complement but also to reduce many of the currently used wet-lab experimental models in an inexpensive manner. They open several new possibilities in the field of personalized medicine. However, a major limitation of these databases is their sheer volume and difficulty in handling them. The *in silico* models, which apply sophisticated algorithms, help in handling voluminous data of different cancer databases, to advance scientific understanding. Experience in computational analysis and specialized computational labs capable of handling big data are necessary to utilize the full potential of these databases. Therefore, a systematic approach is taken to analyse these data and model different outcomes. These can be built based on several approaches such as statistical models, network-based models, and tissue-based models (99). Examples of these models are provided in **Table 2**.

**Table 2:** Web-based resources of *in silico* platforms for modelling cancer.

Main Category	Sub category	*In silico* analysis platforms	Features	Web link	References
**Statistical model**	Gene Expression Models	Ensembl	Annotate genes, computes multiple alignments, predicts regulatory function, and collects disease data. Ensembl tools include BLAST, BLAT, BioMart and the Variant Effect Predictor (VEP)	https://asia.ensembl.org/index.html	(100)
		UCSC Genome Browser	A large genomic data repository with a wide range of tools to align genes, predict regulatory regions etc.	https://genome.ucsc.edu/	(101)
	Pathway Enrichment Models	Kyoto Encyclopedia of Genes and Genomes (KEGG)	Database resource for understanding high-level functions and utilities of the biological system from molecular-level information, especially large-scale molecular datasets generated by genome sequencing and other high-throughput experimental technologies.	https://www.genome.jp/kegg/	(102)
		Gene Ontology (GO)	Computational representation of our current scientific knowledge about the functions of genes	http://geneontology.org/	(103, 104)
**Network-Based models**	Protein interaction networks	Database of Interacting Proteins (DIP)	Catalogues experimentally determined interactions between proteins	https://dip.doe-mbi.ucla.edu/dip/Main.cgi	(105)
	Protein interaction networks	STRING interactome	Models protein interactions based on several parameters including physical, co-expression, co-mentioned etc.	https://string-db.org/	(106)
	Cellular Signaling	Database of Quantitative Cellular Signaling (DQQCS)	Repository of models of signalling pathways.	https://doqcs.ncbs.res.in/	(107)
	Stoichiometric Models of Biochemical Reaction	Kinetic Data of Bio-molecular Interactions Database	A database of experimentally determined kinetic data of protein-protein, protein-nucleic acid, protein-ligand, nucleic acid-ligand binding or reaction events described in the literature.	http://bidd.nus.edu.sg/group/kdbi/kdbi.asp	(108)

A new *in silico* approach to drug development is based on AI (artificial intelligence). Machine learning and deep learning methods are employed to improve the above models (109). These models can automatically access most of the available data and draw a conclusion using the AI approach to design lead compounds, refine existing drugs, validate them on artificial disease models and predict the emergence of drug resistance (110). Some software include modelling options. For e.g., Cytoscape is a free software package that provides user-friendly visualization of these open-source data (111). It may be noted that several of the primary databases also integrate these modelling features or allow the users to model the available data according to their needs. Though we have described these platforms primarily as cancer databases, it must be noted that several of these databases serve a dual role of data repository as well as a platform to analyse these data and model cancer. The field is evolving rapidly, and very soon AI-based platforms will be available as a complete package. These packages may be able to offer a more user friendly approach to analysing the databases and thus, supplementing cancer research. Advanced AI platforms, in combination with superior 3D cancer models, have the potential to replace unnecessary animal experiments as they more human relevant besides being faster, resource saving, effective and reproducible.

## 4 Future Roadmap and Perspective

Presently, there is a need to integrate different human-based interdisciplinary research methodologies from the perspective of a holistic understanding of disease mechanisms, prediction, and treatment. 2D cell culture can still serve as a model for large scale pilot drug screening studies or mechanistic studies until 3D cell culture is made more economical and workable. However, after initial screening, different 3D cell culture models can play a big role in narrowing down potential drugs or to reveal specific molecular insights into biological mechanisms. These techniques promise better and more accurate results than conventional 2D cell culture models. Preliminary analysis using widely available human OMICs data alongside or in prior can help in better predictions and minimise the need for additional laboratory experiments. Simple co-culture experiments using 3D setup can aid groups studying the tumor microenvironment to understand complex interactions of cancer and non-cancer cells, which can be integrated with organ chip or the printed organ model to mimic actual organ with high precision. Publicly available datasets can be used for basic to extensive *in silico* analysis to understand the etiology, design drugs, and predict disease outcomes. For non-computational or wet-lab researchers, several software tools are publicly available and being developed in a way that can easily be handled by them for basic yet important analysis. While few research groups can independently perform and integrate both wet-lab and computational analysis, there is a need for scientists to collaborate and form large interdisciplinary groups, get people from diverse backgrounds under one umbrella to solve difficult and important problems. The use of systems biology approaches by the amalgamation of different human-specific research approaches (by integrating diverse research groups) promises to save time, resources, and provide specificity by reducing errors. Scaling of human biobanks would be vital for aiding human-centric cancer research. Its objective is not only to aid in personalized therapy but also provide human tissues for basic and translational research. Few existing cancer biobanks are listed here (112). With evolving modern *in vitro* culture methodologies, the cancer biobank can provide human cells/tissues to research labs to understand molecular mechanisms, intracellular communications, regulation of microenvironment, besides being used for high-throughput screening and personalized therapeutics. These can potentially more precisely tap into areas and scope of research which was not possible with animal models.

**Figure 1:**
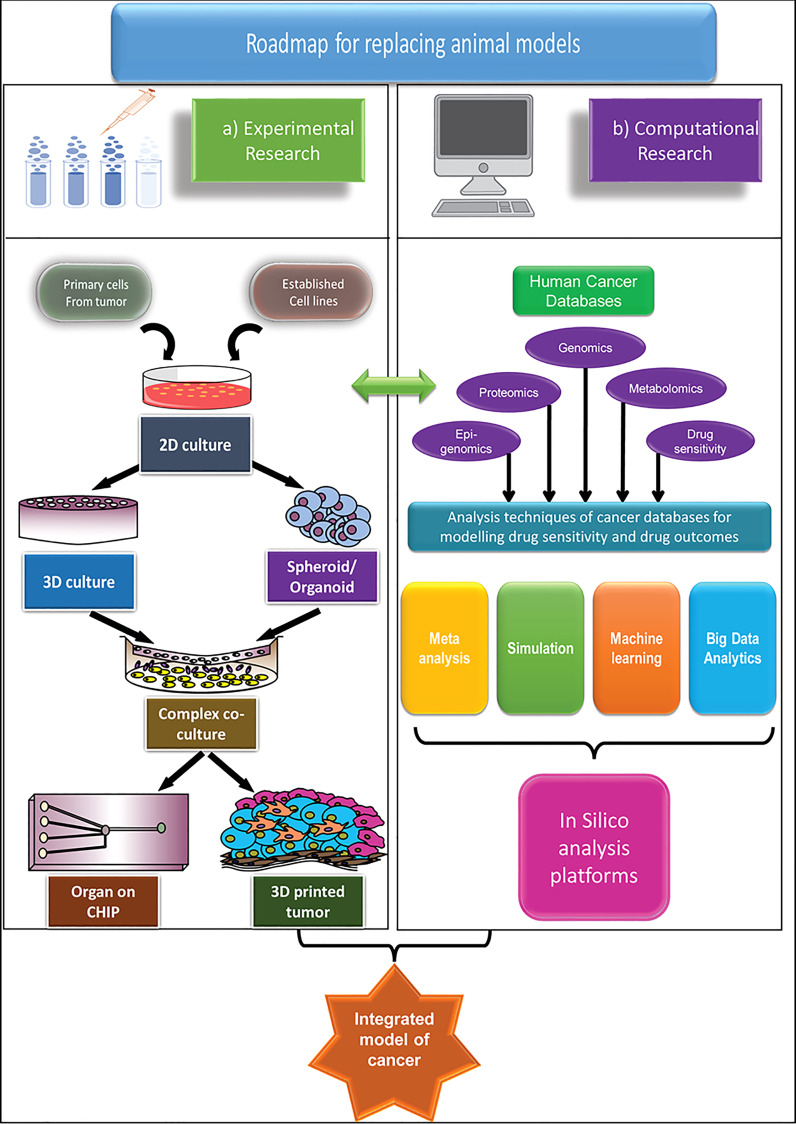
An integrated roadmap to apply advanced human-centric research approaches and potentially replace animal models in cancer research, both for basic and applied research. Two main approaches are considered for replacement of animal models **(A)** Experimental model and **(B)** Computational model. The experimental models are mainly based on the *in vitro* cell culture platforms, which grows in complexity from simple 2D models to the extremely sophisticated organ-on-chip, or 3D printed tumour models to match the specific microenvironment of primary cancer. The computational models take advantage of the different human-based databases and apply different computational methods to model cancer and its outcome. While both methods are advancing significantly, only by combining the two models, we can hope to truly predict the outcome of the cancer therapeutics.

Biobanks represent the core for the development of future human-centric research. Cancer tissue biobanks need both vertical and horizontal expansion. For the latest *in vitro* technologies to be of the highest value, a framework has to be in place which ensures rapid, affordable tissue distribution from the biobanks to places (labs and institutes) that do not have easy access. An increase in collaboration both at the level of institute and nation will aid faster development. Policy changes aiming at expanding the cancer biobanks and making them accessible to wider research institutions, whereby promoting projects involving collaborations with hospitals will enhance and widen the scope of human-centric research. Minimizing extensive paperwork for setting up hospital collaborations without compromising on good research practice would be a welcoming step in this regard. Alongside, development of accessible, more economical and advanced cell culture techniques and availability of user-friendly, free computational tools with increased availability of human tissue/cell repositories, surely has the potential to replace animal-centric models that have not been successful enough for long. The proposed roadmap of the human-centric cancer research models as an alternative to the animal-centric model is demonstrated in **Figure 1**.

We must realize that developing human-centric methods are essential for studying heterogeneous human diseases like cancer, where disease progression depends upon multiple components. With significant advancements in both wet-lab and dry lab technologies, it gives us the opportunity to work with human cells, thereby moving close to understanding the disease. These advanced set-ups will provide greater leads in understanding the disease both from the mechanistic and therapeutic perspective. We envisage that the human-centric advancements will add great value in reducing cancer-related death by adding specificity to treatment against cancer and specifically drug resistance. We need to shift from a cultural reliance on animal models to more human-centric models which certainly seems to be a relevant and an economical substitute. The integration of computational biology with the modern *in vitro* human-based cell culture techniques has the potential to replace the necessity for animal experiments. The merits and demerits of all the different models in cancer research are described in **Table 3**.

**Table 3:** Potential and limitations of different cancer research models (including *in vitro*, *in vivo* and *in silico* models).

Tools in cancer research		Scientific potential	Limitations	Required Infrastructure	Required level of Training	Cost
**Animal Models**	Transgenic (GEM models), Autograft, xenograft and PDX models,	• Controlled environment • Provides 3D tumor architecture • Presence of tumor microenvironment • Genetic manipulation possible • Drug-host interaction	• Anatomical and physiological difference with human • Lack of immune interactions in immunocompromised animals • Difficult and time consuming • Lacking tumor heterogeneity • Different drug-host interaction profile • Costly • Ethical issues and complexitie	• Specialized animal house and animal care facilities including ethical committee approval • Level-2/3 biological safety laboratory • Trained professionals dedicated for animal handling	Very High	Very High
**Conventional non-animal models**	2D cell culture models	• Easy to perform • Drug testing is easy and effective • Considerably less ethical issues than animals • Genetic manipulation possible	• Dissimilarity with 3D architecture of original tumor • Absence of tumor microenvironment • Lack of immune interactions • Lack of tumor heterogeneity • Absence of drug-host interactions • Cannot mimic the blood flow.	• Level-2/3 biological safety Laboratory • Cell culture experience	Low	Low
	Scaffold based 3D cell culture models	• Can provide 3D tumor architecture • Can provide a platform to create Tumour microenvironment • Genetic manipulation possible • Drug testing is more effective. • Considerably less ethical issues than animals.	• Tumor microenvironment is represented simplistically. • Lack of immune interactions • Lack of tumor heterogeneity • Absence of drug-host interactions. • Cannot mimic the blood flow.	• Scaffold fabrication facility • Level-2/3 biological safety Laboratory • Trained professionals for animal cell culture	Moderate	Moderate
**Emerging human cell based models**	Spheroid, Organoid, models	• Can provide a better 3D tumor architecture • Can mimic Tumor microenvironment • Can recreate tumor heterogeneity • Genetic manipulation possible • Drug testing is more effective • Considerably Less ethical issues than animals	• Lack of immune interactions • Absence of drug-host interactions • Cannot mimic the blood flow.	• Level-2/3 biological safety Laboratory • Trained professionals for organoid/spheroid culture	Moderate	Moderate
	Organ on chip, tumor on chip	• Can provide host like 3D tumor architecture • Can mimic Tumor microenvironment which almost resembles a human tumor. • Can recreate tumor heterogeneity • Immune interaction modelling is possible • Can mimic the blood flow. • Genetic manipulation possible • Drug testing is easy and more effective • Can mimic drug-host interactions • Considerable less ethical issues	• Immune interactions are not similar to the host • Drug-host interactions are not exactly similar to the host	• Fabrication facilityImmune interactions are not similar to the hostLevel-2/3 biological safety LaboratoryImmune interactions are not similar to the host>Trained professionals for animal cell culture/organoid/spheroid culture.	High	High
**Databases**	Genetic, epigenetic, proteomic and metabolomics databases	• Some contains real patient data. • Can predict drug effectiveness in human • Can predict adverse/favorable outcomes of mutations, therapies, and protein expression. • Can help in drug discovery • Low cost • No ethical issues.	• Requires training • Needs huge datasets to address racial/geographical variations of population • Requires experimental validation after computational prediction.	• Adequate computing power/software • Trained professionals for computational analysis	Moderate	Low

Overall, the whole community including academic and industrial partners as well as their regulators need to show more confidence in using human-based advanced models and explore avenues to better exploit these models for improved drug-predictability, leading to lesser drug attrition, faster and efficient drug development, leading to higher clinical success rates. Collaborative efforts of cancer biologists, computational biologists, and data mining specialists can lead to a better interpretation of these data. Government policy makers and universities should promote such collaborative endeavours by opening new interdisciplinary centers. Funding must be raised from numerous sources to support and increase the reach of such platforms. Although several agencies and charitable institutes are supporting and encouraging funding for non-animal projects, a funding thrust from government and more private agencies can significantly promote the usage and validations using advanced *in vitro* models.

## Author Contributions

AP: Conceptualization, literature search, writing, proof reading, framework design. SD: Literature search, writing, conceptualization, figure drawing, proof reading; CD: Literature search, writing, conceptualization, proof reading; MM: Conceptualization, Proof Reading. All authors contributed to the article and approved the submitted version.

## Funding

The review article receives funding from the “Human Society International”.

## Conflict of Interest

The authors declare that the research was conducted in the absence of any commercial or financial relationships that could be construed as a potential conflict of interest.

## Publisher’s Note

All claims expressed in this article are solely those of the authors and do not necessarily represent those of their affiliated organizations, or those of the publisher, the editors and the reviewers. Any product that may be evaluated in this article, or claim that may be made by its manufacturer, is not guaranteed or endorsed by the publisher.
